# Supplementary material for: Medications for opioid use disorder among pregnant women referred by criminal justice agencies before and after Medicaid expansion: A retrospective study of admissions to treatment centers in the United States
Source: PLoS Med. 2020 May 18;17(5):e1003119. doi: 10.1371/journal.pmed.1003119 (PMC7233523; doi:10.1371/journal.pmed.1003119)
Supplement: S1 Text — (DOCX) [file pmed.1003119.s006.docx]

**S1 Text.** Opioid use disorder treatment among pregnant women involved in the criminal justice system.

Tyler Winkelman, Hennepin Healthcare

Becky Ford, Hennepin Healthcare

Rebecca Shlafer, University of Minnesota

Stephen Patrick, Vanderbilt University

**Dataset:** TEDS-A

**Years:** Would be great if we could go back to 1990, but early 2000s would be fine

**Restrictions:** Pregnant women, Sub1 = heroin, non-prescription methadone, or other opiates and synthetics

**Key independent variable:** Primary source of referral

**Primary dependent variable:** Medicaid-assisted therapy part of patient’s treatment plan

Question 1. What are the characteristics of pregnant women with opioid use disorders by justice-involved status?

Demographics available in TEDS-A

1. Age
2. Race/ethnicity
3. Marital status (Not included due to large amount of missing data, >25%)
4. Education
5. Employment
6. Living arrangement/homeless (Not included due to large amount of missing data, >15%)
7. Census region/division based on FIPS codes
8. Treatment facility type

Question 2. How has justice involvement among pregnant women with opioid use disorders changed over time?

Question 3: How has receipt of MAT changed over time among pregnant women with OUD by justice involvement status? How do current rates of MAT compare between groups?

Question 3b: How has receipt of MAT changed over time among pregnant women referred from probation vs. court vs. other? (Question 3b was not pursued due to sample size)

- Unadjusted and Adjusted for characteristics defined in Question 1
