# Supplementary material for: Medications for opioid use disorder among pregnant women referred by criminal justice agencies before and after Medicaid expansion: A retrospective study of admissions to treatment centers in the United States
Source: PLoS Med. 2020 May 18;17(5):e1003119. doi: 10.1371/journal.pmed.1003119 (PMC7233523; doi:10.1371/journal.pmed.1003119)
Supplement: Attachment — Submitted filename: 2020.02.24.Response to Reviewers.pdf [file pmed.1003119.s007.pdf]

Response to Reviewers: PMEDICINE-D-19-03648

	Editor/Reviewer Comment	Response	Page/Line
1	The Academic Editor raised a point and we would ask you to discuss in the manuscript: The Medicaid result is quite strong. However, the other result about criminal justice referral is less strong, because there are potentially unobserved attributes of people referred by one mechanism or another that are also correlated with their receipt of medications for OUD. It is not possible to control for all of those things, so I worry about the issue of endogeneity (or confounding) there.	We agree there are likely many factors, beyond criminal justice involvement itself, that influence why those referred by criminal justice agencies received medications for OUD less often than those referred by other sources. We have added a discussion of potential mediators and/or confounders in our limitations section.<br><br>In our opinion, these alternative mechanisms still lead to the conclusion that pregnant women referred to treatment by criminal justice agencies systematically receive inferior treatment compared to those referred by other sources. For example, in our experience, criminal justice agencies frequently refer to specific treatment centers because of state or county contracts, whereas those with individual or other referrals are more likely to go a wider number of facilities. If criminal justice agencies refer patients to treatment centers that do not provide medications for OUD, then these patients will systematically not receive medications. We cannot control for these types of mediators that are likely present and help to explain the disparity between referrals from criminal justice agencies vs. other sources:<br><br>*"Fifth, our data do not allow us to isolate why pregnant women referred by criminal justice agencies receive medications for OUD less often than those referred by other sources. For example, it is possible that those referred by criminal justice agencies are referred to different facilities or that those with criminal justice involvement refuse medications due conditions of community supervision or mistrust of the health care system."*	p.15, ln 439-444
2	Please revise your title according to PLOS Medicine's style. Your title must be nondeclarative and not a question. It should begin with main concept if possible. "Effect of" should be used only if causality can be inferred, i.e., for an RCT. Please place the study design ("A randomized controlled trial," "A retrospective study," "A modelling study," etc.) in the subtitle (ie, after a colon). Also please add the country in which the study is set.	We have changed the title to fit with Plos Med's style guidelines. It now starts with "Medications for opioid use disorder," and includes study design and the country in which the data were collected in the subtitle.<br><br>*"Medications for opioid use disorder among pregnant women referred by criminal justice agencies before and after Medicaid expansion: A retrospective study of admissions to treatment centers in the United States"*	Cover page p. 1, ln 1-3

3	Abstract- Please add summary demographic information of the women included in the study; please add p values (and elsewhere I tables / text) where 95% Cis are given; the final sentence of the Methods and Findings section should include a description of the studies limitations.	Summary demographic information has been added to the Methods and Findings section of the abstract. P-values were also added in the abstract and elsewhere where CIs are listed.<br><br>*"Women in the sample were predominantly aged 18-34, White non-Hispanic, completed high school, and were not employed."*<br><br>We added the following limitations to the Methods and Findings in the abstract:<br><br>*"This study includes encounters at treatment centers only and does not encompass buprenorphine that is prescribed in ambulatory care settings or in prisons or jails."*	Abstract p. 2, ln 36-44
4	Data – the URL provided returns 'page not found'. PLOS Medicine requires that the de-identified data underlying the specific results in a published article be made available, without restrictions on access, in a public repository or as Supporting Information at the time of article publication, provided it is legal and ethical to do so.	We have confirmed that the following URL leads to a list of all available data (currently 1992-2017): https://www.datafiles.samhsa.gov/study-series/treatment-episode-data-set-admissions-teds-nid13518	
5	At this stage, we ask that you include a short, non-technical Author Summary of your research to make findings accessible to a wide audience that includes both scientists and non-scientists. The Author Summary should immediately follow the Abstract in your revised manuscript. This text is subject to editorial change and should be distinct from the scientific abstract.<br><br>We ask authors to provide 2-3 single sentence bullet points for each of the following questions. Bullet points should be objective, brief, succinct, specific, accurate, and avoid technical language.<br><br>Why Was This Study Done? Authors should reflect on what was known about the topic before the research was published and why the research was needed.<br><br>What Did the Researchers Do and Find? Authors should briefly describe the study design that was used and the study's major findings. Do include the headline numbers from the study, such as the sample size and key findings. What Do These Findings	Why was this study done?<br>• There has been a four-fold increase in the number of pregnant women with opioid use disorder (OUD). Medications such as methadone and buprenorphine are standard of care for opioid use disorder and are recommended during pregnancy, but only 50% of pregnant women receive such medication.<br>• Pregnant women with OUD who are involved in the criminal justice system are at high risk of poor outcomes and data regarding the use of medications for OUD in this population are limited.<br><br>What did the researchers find?<br>• From 1992-2017, pregnant women in the United States who were referred to treatment for opioid use disorder by a criminal justice agency (vs. other referral sources) were half as likely to receive medication as part of their treatment plan.	p. 3, ln 65-91

	Mean? Authors should reflect on the new knowledge generated by the research and the implications for practice, research, policy, or public health. Authors should also consider how the interpretation of the study's findings may be affected by the study limitations.	• After implementation of the Affordable Care Act's Medicaid expansion, medication for OUD increased significantly more among pregnant women referred to treatment by criminal justice agencies in Medicaid expansion states compared with non-expansion states.<br><br>What do these findings mean?<br>• Pregnant women referred to treatment for OUD by criminal justice agencies were consistently less likely to receive evidence-based treatment, which increases their risk of overdose and poor maternal and neonatal outcomes.<br>• Improving access to Medicaid for justice-involved individuals may increase the rate at which pregnant women receive evidence-based treatment for OUD.	
6	Please avoid bold font in main text for emphasis	Bold font has been removed from the text. Section headings, tables, and figures still contain bolded font.	
7	Did your study have a prospective protocol or analysis plan? Please state this (either way) early in the Methods section.	See below	
8	a) If a prospective analysis plan (from your funding proposal, IRB or other ethics committee submission, study protocol, or other planning document written before analyzing the data) was used in designing the study, please include the relevant prospectively written document with your revised manuscript as a Supporting Information file to be published alongside your study, and cite it in the Methods section. A legend for this file should be included at the end of your manuscript.	We have included the following description of our analysis plan in the statistical analysis section:<br><br>*"We pre-specified our analysis plan to examine trends in medications for OUD over time among pregnant women who were referred by criminal justice agencies or other sources. After these initial analyses, we conducted an additional difference-in-differences analysis using previously described specifications to investigate the extent to which the increase in medications for OUD after 2014 was associated with Medicaid expansion and hypothesized that this increase would largely be explained by Medicaid expansion."*	p. 7, ln 206-211
9	Please ensure that the study is reported according to the [STROBE] guideline, and include the completed [STROBE or other] checklist as Supporting Information. When completing the checklist, please use section and paragraph numbers, rather than page numbers. Please add the following statement, or similar, to the Methods: "This study is reported as per the Strengthening the	We have confirmed that we have reported in accordance with the STROBE checklist for cross-sectional studies. This sentence has been updated in the methods section and a copy of the checklist is now attached.	p. 8, ln 241-243

	Reporting of Observational Studies in Epidemiology (STROBE) guideline (S1 Checklist)."		
**Statistical reviewer**			
10	From what I understand there was a big political difference in the two types of states (expansion vs non- expansion). From what I understand from natural experiments, to get robust causal conclusions you'd want the two groups to be comparable at the time the change is made (perhaps after an adjustment through matching). In this case the prescription behaviour seems rather different both before and after the Medicaid expansion. Is there strong potential for confounding in this? Would matching by other characteristics of states be possible? Having said all that, the results do look fairly convincing in terms of the trajectory before and after expansion.	The difference-in-differences approach to analyzing observational data allows for time invariant unobserved differences as long as both groups have parallel trajectories prior to the change (in this case, Medicaid expansion). A difference-in-differences approach is generally the preferred method for conducting analyses related to the ACA's Medicaid expansion (*See citations below*). We agree that states that expanded Medicaid after the passage of the ACA are politically different than those that did not, but these differences are present both before and after the passage of the ACA and, therefore, should not influence our difference-in-differences estimates. Finally, there is some evidence that suggests matching introduces additional bias into difference-in-differences estimates<br>(Daw JR, Hatfield LA. Matching in difference-in-differences: between a rock and a hard place. Health Serv Res. 2018;53(6):4111-4117).<br><br>Sommers BD, et al. Changes in self-reported insurance coverage, access to care, and health under the Affordable Care Act. JAMA. 2015;314(4):366-374.<br><br>Miller S, Wherry LR. Health and access to care during the first 2 years of the ACA Medicaid expansions. NEJM. 2017;376(10):947-956)<br><br>Saloner B, et al. Changes in buprenorphine-naloxone and opioid pain reliever prescriptions after the Affordable Care Act medicaid expansion. JAMA Netw Open. 2018;1(4):e181588.<br><br>Mojtabai R. The Affordable Care Act and Opioid Agonist Therapy for Opioid Use Disorder. Psychiatr Serv. 2019;70(7):617-620.	
11	Abstract - would be useful to have effect sizes + CIs for the difference between the referral paths rather than just CIs for each arm separately.	The abstract now refers to the percentage of pregnant women with referrals from criminal justice agencies, and how those percentages differ from those with individual and other referrals:	Abstract p. 2, ln 34-41

		"We used multiple logistic regression and difference-in-differences methods to assess trends in medications for OUD by referral source. Women in the sample were predominantly aged 18-34, White non-Hispanic, high school graduates, and not employed. Over the study period, 26.3% (95% CI 25.7-27.0) of pregnant women referred by the criminal justice system received medications for OUD, which was significantly less than those with individual referrals (adjusted rate ratio [ARR] 0.45, 95% CI: 0.43, 0.46; P<.001) or those referred from other sources (ARR: 0.51, 95% CI: 0.50, 0.53; P<.001)."	
12	Abstract - number of expansion and non-expansion states?	There are 32 expansion states (including the District of Columbia) and 18 non-expansion states included in the sample (Florida was excluded). This information has been added to the abstract.	Abstract p. 2, ln 42-43
13	Line 83 - is it possible to link admissions of the same individual? If so, are there many individuals that are seen multiple times? If so, did the authors consider using a method that would allow for correlation within admissions from the same individual?	It is not possible to link admissions, and there may be multiple admissions for the same individual. According to the TEDS-A codebook: *Many states submit records that include multiple admissions for the same client. Therefore, any statistics derived from the data will represent admissions, not clients. It is possible for clients to have multiple initial admissions within a state—and even within providers that have multiple treatment sites within the state. A few states uniquely identify clients at the state-level; several more are attempting to achieve this level of client identification. The TEDS provides a good national snapshot of what is seen at admission to treatment, but is currently unable to follow individual clients through a sequence of treatment episodes.*<br><br>We have added an explanation to the methods section.<br><br>*"TEDS-A does not contain identifiable information, so it is not possible to links admissions for the same individual."*	p. 5, 146-147
14	Line 144/145 - I understand the rationale for the washout period, but did this make a large difference to the results? Would be interesting to include 2014 to see (unless the pre-specified analysis plan excluded 2014).	We did not previously estimate differences that included 2014 data, because it is common in ACA-related analyses to exclude 2014. To address the reviewer's question, we did re-estimate our models to incorporate the wash-out period. As expected, the association between medications for OUD and expansion was similar, but reduced due to partial implementation (Difference-in-difference estimate: 8.7 percentages points (95% CI: 0.1-17.4;	

		P=.049) compared to 12.0 percentage points (95% CI: 1.0, 23.0; *P*=.03) when 2014 was excluded).	
15	Statistical analysis - was there any missing demographic data, or were individuals only included with complete data? Any issues of representativeness in that case?	Two demographic variables outlined in our prospective analysis plan, marriage status and living arrangement, were greater than 10% missing and we ultimately chose to exclude these variables from analysis because of concerns about data quality. 2.7% of admissions were missing a referral source and these were excluded. Of the remaining admission, <3% contained missing data. Overall, <5% of cases were excluded due to missing data. We added the following statement to the methods:<br><br>"*Admissions with missing referral source were excluded from all analyses (2.7%), while admissions with missing covariates were excluded from regression models.*"	p. 6, ln 166-168
16	Line 156 - p-values are mentioned but no p-values are given, so I don't think this sentence is needed.	The editor has asked us to provide p-values in Table 1 and wherever CIs are given so we have kept this sentence in the manuscript.	
17	Would be interesting to see whether same results for expansion vs non-expansion would be the case for the other referral paths too - why just looking at prison?	We generated difference-in-differences estimates for each referral source and included the following text:<br><br>*"Per reviewer request, we also compared our difference-in-differences estimate among pregnant women referred by criminal justice agencies to women referred by other sources."*<br><br>*"The difference-in-differences estimate among pregnant women referred by criminal justice agencies was similar to individual referrals (Adjusted DID 11.9 pp, 95% CI 2.3, 21.4; P=.02) and other referral sources (Adjusted DID 13.4 pp, 95% CI -1.3, 28.0; P=.07)."*<br><br>*"Although the ACA did not alter Medicaid coverage during pregnancy[31], we found that the ACA's Medicaid expansion was associated with a significant increase in medications for OUD among pregnant women with a criminal justice agency or individual referral in expansion states compared to non-expansion states."*	p. 8, ln 237-239<br><br>p. 13, ln 350-352<br><br>p. 14, ln 402-405
Reviewer 2			

18	The authors found that a large proportion of women did not receive standard of care treatment with medications, and furthermore, that those referred from the criminal justice system received even less appropriate treatment with medications. The authors also found that, not surprisingly, receipt of medications was higher in Medicaid expansion states.	N/A	
19	The authors did not include Florida- would it be possible to look at the Florida data and see, at least if the data that is available is consistent with the rest of the country? If not, how so? The state is a large one and it would be re-assuring if the data there were at least consistent.	During the data cleaning phase, we examined frequencies for each state by year. We noticed that Florida had almost double the number of admissions in 2012 as they did in 2011 or 2013. We communicated with SAMHSA about abnormalities in the TEDS data from Florida. Here is their response: "*The state of Florida reported in the past that they made many changes in their data during that time. They reported that it is nearly impossible to compare data across those years. Florida documented reporting issues due to personnel changes and system changes. The CY2012 had fewer records due to a change in how data for TEDS were extracted and the loss of several private providers. The programmer at that time worked on getting their data close to the TEDS requirements as possible.*" Because of this, we do not feel it is appropriate to report data from Florida.<br><br>During the full study period (1992-2017), 24.8% of pregnant women in Florida who were referred to treatment by criminal justice agencies received MOUD, compared to 25.8% of those referred by other sources and 62.7% of individual referrals.	
**Reviewer 3**			
20	Thank you for the opportunity to review this manuscript. This is a novel study on an important and overlooked topic that, at its core, seems to suggest discrimination against pregnant people referred for treatment from criminal justice agencies, that they receive worse treatment than those without. The statistical approach is a compelling one to highlight this problem. It's a promising paper with an innovative approach with a variable that receives little attention when it comes to pregnant people, criminal justice system involvement.	Thank you	
21	This paper would be greatly strengthened by providing more detail and clarity on a number of methodological/definitional fronts to better orient the reader and contextualize the meaning of and limits of the data we can know from this data source; specific suggestions on where to do this are outlined below.	We have responded to each comment below. We have provided more detail using the TEDS-A codebook, as well as the reporting instructions given to treatment facilities.<br><br>According the TEDS-A codebook, a criminal justice referral	

		means "*Court/criminal justice referral/DUI/DWI: Any police official, judge, prosecutor, probation officer, or other person affiliated with a federal, state, or county judicial system. Includes referral by a court for DWI/DUI, clients referred in lieu of or for deferred prosecution, or during pretrial release, or before or after official adjudication. Includes clients on pre-parole, pre-release, work or home furlough, or TASC. Client need not be officially designated as 'on parole'. Includes clients referred through civil commitment."*	
22	Title, abstract, and throughout uses the term "the criminal justice system." This makes it sound like a monolithic entity, which it is not, as the authors are well aware. In calling it that, it's confusing to know whether referrals came from jails, prisons, drug courts, probation, parole. This monolithic descriptor makes it seem like they are all the same, when they all have very different processes. This should be clarified and defined up front in the abstract and the intro. It takes going deep into the methods section to realize that this actually does not include prisons or jails, which is important to explain up front	We added more information about referrals and settings of treatment. These additions have been made throughout the paper (title, abstract, introduction, methods, discussion).<br><br>*"While pregnant women with OUD are increasingly referred to treatment by criminal justice agencies, such as court-mandated treatment or treatment as a condition of probation/parole, there are limited data on trends in medications for OUD for this population[7,8]."*<br><br>*"Furthermore, TEDS-A contains information from admissions to treatment centers that accept public funding, and it does not include information about treatment that occurs in other facility types (e.g., primary care offices, in health clinics inside jails and prisons, treatment centers that do not accept public funding)."*<br><br>*"Criminal justice agency referrals included referrals to treatment centers from police, probation officers, judges, prosecutors, DUI/DWI court, and parole boards. Criminal justice referrals do not include treatment received within a correctional facility."*<br><br>*"Furthermore, TEDS-A only includes data from treatment centers in the community, and individuals who are receiving treatment inside of a corrections facility (prison or jail) are not included"*	Page 4, line 109-112<br><br>p. 5, ln 147-150<br><br>p. 6, ln 174-177<br><br>Page 15, ln 431-433
22	Perhaps saying criminal justice system agencies is better (as in line 57), and then being very explicit in the introduction and discussion that these are community based CJS agencies, or some other way of saying that this does not include jails and prisons.	We thank the reviewer for pointing out that "the criminal justice system" may lead readers to believe that we are referring to treatment that occurs in jails and prisons, rather than referrals to outside treatment centers. We have changed all references to referrals to treatment from "the criminal justice system" to "criminal justice agencies." We have reserved use of the term	

		"criminal justice system" to instances when we are discussing the system at large, rather than just referrals.	
24	Abstract-Lines 26-27-could use some more clarity in the first sentence. "data of pregnant women in the United States who reported opioids as their primary reason for treatment (N=131,838)." It's confusing not knowing the source of the data and opioids as primary reason for treatment from what kind of provider or agency. . . While the manuscript will get into more detail of course, this first sentence confused me as to how various data sources were defined. It's also confusing without that context what these women would be referred for if not for M-OUD. Are they just being referred for prenatal care? Or is it drug treatment referrals you are talking about and they just get behavioral treatment? Just mentioning that the data come from a national survey of admissions to substance treatment facilities would be orienting for the reader.	We changed the abstract sentence to clarify who is represented in the data. It now specifies that our sample is pregnant women admitted to treatment centers for opioid use, and that the data source is a national survey of treatment centers. We also added a sentence to the first paragraph of the introduction explaining what treatment would typically look like it if did not include medication.<br><br>Abstract: *"We conducted a serial, cross-sectional analysis using 1992-2017 data from pregnant women admitted to treatment facilities for opioid use disorder using a national survey of substance use treatment facilities in the United States (N=131,838)."*<br><br>Intro: *"Treatment of OUD with medication has consistently been shown to improve pregnancy outcomes and reduce the risk of overdose-related death [4–6] compared with non-medication based treatment, which would typically include individual/group counseling services, withdrawal management, or referrals to other social services."*	p. 2, ln 32-34<br><br>p. 4, ln 97-101
25	Intro/Lines 60-66: This part of the intro needs more explanation as to what you mean by 'pregnant women referred by the criminal justice system.' Does this mean people who are not in custody at the time of the referral? Which kinds of agencies? How do CJS agency referrals for MOUD even happen? Are they court mandated referrals? The "referral" aspect is the crux of the study, but I think it could use a little more specificity as to how/what this really means.	Referrals are made through a heterogeneous process that varies by jurisdiction. TEDS does not provide information on the precise mechanism through which each referral is made. Based on our clinical experience, the source of the referral is indicated on intake forms at treatment centers. Referrals through criminal justice agencies can be triggered through a variety of mechanisms, including court mandate or suggestion by a probation officer.<br><br>*"While pregnant women with OUD are increasingly referred to treatment by criminal justice agencies, such as court-mandated treatment or treatment as a condition of probation/parole, there are limited data on trends in medications for OUD for this population[7,8]."*	p. 4, ln 109-112
26	Relatedly, it would also be helpful to contextualize what "other sources" means. It likewise sounds monolithic but likely is not, and would be useful to elaborate more on what you mean by this,	We have added a couple of examples for each referral source in the introduction.	p. 5, ln 1225-128

	in contradistinction to criminal justice agencies. While the methods section gets into this more, the intro needs some of this overall context (though not as much specificity as in the methods)— also because once you read the methods you see that "the criminal justice system" as available in the database and therefore as defined in this study actually does not include jails or prisons.	*"We compared pregnant women referred to treatment facilities by criminal justice agencies to those with individual referrals (e.g., self-referral to treatment, referred by a friend or family member) and those referred from other sources (e.g., referral from a health care provider or employer)."*	
27	Intro would also benefit from some information on trends in women's involvement in criminal justice system agencies that are being described—pregnant women data would be ideal, but I suspect this is not available.	We added additional information about trends in women's involvement in the criminal justice system:<br><br>*"Since 1980, the incarceration rate among women increased two times faster compared with men [9]."*	p. 4, ln 106-107
28	78-85: Clarify that this is a survey done annually (if indeed it is). Perhaps I missed this mention, but it's worth saying here in the methods that it is an annual survey.	Treatment centers report annually. This has been added to the methods.<br><br>"...an <u>annual</u> national survey of substance treatment facility admissions …"	p. 5, ln 138
29	87-91- Please explain how this survey asks about/records/verifies pregnancy status, so we know how the sample population is ascertained.	States are instructed to indicate if a female patient was pregnant at the time of admission. SAMSHA does not specify how treatment centers should obtain this information. From our clinical experience, we know that pregnancy status is usually listed in the referral when it comes from a criminal justice agency or health care organization. Women are also often asked to self-report their pregnancy status during the intake process. Many treatment centers require pregnancy tests for women entering treatment, although we do not know how widespread this practice is.<br><br>*"Data elements are obtained from information included in the referral itself (e.g., information provided by a criminal just agency or health care provider at the time of referral) and during an interview that is conducted with the patient at the time of intake to the treatment facility."*	p. 5, ln 141-144
30	94-95: Could use a brief few words that 'referral for treatment' could be just for behavioral treatment—to underscore that medication treatment is something separate that is not necessarily a given with a referral.	We added a sentence to the *Key dependent variable* section of the Methods.<br><br>*"Referrals to treatment do not necessarily ensure an individual will receive medication for OUD and could, instead, result in counseling or other behavioral treatment only."*	p. 6, ln 188-189

31	118-123: How are these variables assessed by the person reporting? Do we know? Is it just "reported race/ethnicity" etc.? Also, how does this survey distinguish for-profit facilities that do not accept insurance? The limitations section mentions something about this, but in explaining the database to the readers in the methods, some of this information is helpful up front.	Demographic information is typically obtained through patient self-report. We have added this information to the methods section. We have also added information to the methods about which treatment centers are mandated to report to SAMSHA and which are not.<br><br>*"Data elements are obtained from information included in the referral itself (e.g., information provided by a criminal just agency or health care provider at the time of referral) and during an interview that is conducted with the patient at the time of intake to the treatment facility."*<br><br>*"Furthermore, TEDS-A contains information from admissions to treatment centers that accept public funding, and it does not include information about treatment that occurs in other facility types (e.g., primary care offices, in health clinics inside jails and prisons, treatment centers that do not accept public funding)."*	p. 5, ln 141-144<br><br>p. 5, ln 147-150
32	Discussion: This section too needs a reminder that this is talking about people in the community who are referred by criminal justice system agencies-- that this is not about incarcerated people.	We have changed the first sentence of the Discussion to remind readers that we are talking about treatment facilities. We have also added a sentence to the *Limitations* reminding readers that TEDS-A does not include treatment that may occur inside of corrections facilities.<br><br>*"From 1992-2017, pregnant women with OUD referred to substance use treatment facilities by criminal justice agencies were consistently less likely to receive medications for OUD than pregnant women referred by other sources."*<br><br>Limitation: *"Furthermore, TEDS-A only includes data from treatment centers in the community, and individuals who are receiving treatment inside of a correctional facility (prison or jail) are not included."*	p. 13, ln 362-364<br><br>p. 15, ln 431-433
33	Somewhere in the discussion it's also worth note, even as the authors note that the database does not distinguish methadone vs. buprenorphine, that buprenorphine did not become as widely used in pregnancy until after the results of the MOTHER trial were published in 2012. Is there some way that this might come into play?	It's possible that the MOTHER study led to an increase in the use of buprenorphine among pregnant women, in addition to a general push in the medical community to increase buprenorphine use for OUD around this time. However, it is unlikely that the MOTHER study would influence expansion and non-expansion states differently and, thus, should not interfere with our analytic	

		approach. Furthermore, we do not find substantial changes in medications for OUD until the ACA's key provisions were fully implemented in 2015.	
34	The discussion section should acknowledge that there may be other reasons pregnant people are not on M- OUD—including that they decline medication treatment. This study could not assess for people who voluntarily do not want to me on medication. And people referred by CJS agencies are different than those from other sources, eg. may have experienced more coercion, mistrust etc., and this may account for some of the difference (maybe CJS referred people were offered medications but were more likely to decline) which this study cannot measure.	We agree that Medicaid expansion status is not the only explanation why pregnant women with referrals from criminal justice agencies receive medication for OUD less often than those with referrals from other sources, and we appreciate the reviewers' possible alternative explanations. These alternative explanations still highlight the need to improve access to medication for OUD for this particularly vulnerable population. We have added a section to our limitations section outlining these alternatives:<br><br>*"Fifth, our data do not allow us to infer why pregnant women referred by criminal justice agencies receive medications for OUD less often than those referred by other sources. For example, it is possible that those referred by criminal justice agencies are referred to different facilities due to state/county contracts with treatment centers, or that those with criminal justice involvement refuse medications due conditions of community supervision or mistrust of the healthcare system."*	p. 15, ln 439-444
35	We cannot assume that people who had referrals by self or other did not have CJS involvement. They certainly might, it's just that a CJS agency wasn't the one who referred them to treatment. So can we really isolate this as CJS effect? Maybe people who were told by a CJS agency that they had to be in treatment didn't want to accept medication treatment offered to them, whereas women who referred themselves, even if they might have also had CJS involvement, would be more likely to be open to medication. So it's not just that as stated in lines 315-316 this study can't account for people with CJS involvement who were not referred, it's also that people who self referred may also have CJS involvement, it's just not mandated/part of the referral.	We appreciate the reviewer's point. There are certainly women with criminal justice involvement who self-refer to treatment, and there are cases when courts will allow people to self-refer to avoid a DUI/DWI conviction. We have added a statement on this topic to the limitations section.<br><br>*"Many individuals with OUD have involvement with the criminal justice system who are not linked to treatment, and some individuals with criminal justice involvement will self-refer to treatment without an actual referral from a criminal justice agency."*	p. 15, ln 437-439
