# Supplementary material for: Medications for opioid use disorder among pregnant women referred by criminal justice agencies before and after Medicaid expansion: A retrospective study of admissions to treatment centers in the United States
Source: PLoS Med. 2020 May 18;17(5):e1003119. doi: 10.1371/journal.pmed.1003119 (PMC7233523; doi:10.1371/journal.pmed.1003119)
Supplement: Attachment — Submitted filename: 2020.04.03.Response to Reviewers.docx [file pmed.1003119.s008.docx]

Response to Reviewers: PMEDICINE-D-19-03648

| Editor/Reviewer Comment | Response | Page/Line |
| --- | --- | --- |
| We ask every co-author listed on the manuscript to fill in a contributing author statement. If any of the co-authors have not filled in the statement, we will remind them to do so when the paper is revised. If all statements are not completed in a timely fashion this could hold up the re-review process. Should there be a problem getting one of your co-authors to fill in a statement we will be in contact. YOU MUST NOT ADD OR REMOVE AUTHORS UNLESS YOU HAVE ALERTED THE EDITOR HANDLING THE MANUSCRIPT TO THE CHANGE AND THEY SPECIFICALLY HAVE AGREED TO IT. | Completed | N/A |
| Please ensure that the paper adheres to the PLOS Data Availability Policy | All data are publicly available from the Substance Abuse & Mental Heath Data Archive: https://www.datafiles.samhsa.gov/study-series/treatment-episode-data-set-admissions-teds-nid13518 |  |
| Abstract – please add summary demographic information, including mean age and also is it possible to say which cities or is it truly nationwide (this would also be helpful in the main text? | In TEDS-A, age is listed as a categorical variable instead of continuous, so we are not able to calculate mean age. Most cases (63.3%) are in the 18-24 or 25-29 category, and we have included this in the abstract.  *"Women in the sample were predominantly aged 18-29 (63.3%)…"*  This sample is truly nationwide, as every state in the US is required to report on admissions to treatment centers. | ln 30 |
| Abstract: Can you please add a sentence of limitations as the final sentence of the ‘Methods and Findings section’, or at least be more explicit by starting that sentence with ‘Limitations of our study are……’ | We now include a clause at the beginning of the limitations section of the abstract:  *“Limitations of this study include encounters that are at treatment centers only and that do not encompass buprenorphine prescribed in ambulatory care settings, prisons, or jails.”* | ln 38-40 |
| Where p values are for example .2, please write as 0.2 | This change has been made in the abstract and throughout the paper. |  |
| STROBE checklist – Please amend your checklist as it currently does not appear to have sections and paragraphs. When completing the checklist, please use section and paragraph numbers, rather than page numbers. | A STROBE checklist with page numbers, sections and paragraph numbers has been attached. |  |
| Reviewer 1 |  |  |
| Thank you to the author for addressing my previous comments well. I have no further issues to raise. | Thank you. |  |
| Reviewer 3 |  |  |
| The authors have an outstanding job revising this manuscript and responding to my initial review. The article provides important insights and I recommend this article for publication. | Thank you. |  |
| Line 325- consider adding a citation to this new publication, which describes use of medications for OUD for pregnant and postpartum women in jails and prisons: https://www.ncbi.nlm.nih.gov/pubmed/32141128 | We thank the reviewer for pointing out this very relevant and timely reference. We have added it to the manuscript (Reference #27), and we have updated the reference list. | ln 325 |
| Line 353- add "in" between early and pregnancy | Thank you. This line was not clear, and we edited for clarity:   *“Thus, pregnant women may be more likely to* ***start their*** *pregnancy, and subsequently treatment, with insurance.”* | ln 370 |
| Thank you for the opportunity to review this revised manuscript. | Thank you for your insightful review. |  |
